# Health Resorts

**Published:** 1874-03

**Authors:** 


### HEALTH RESORTS.

### VALLEY OF THE YELLOWSTONE, WYOMING TERRITORY.

As a place of RESORT FOR INVALIDS, the Yellowstone Valley, on account of its pure and exhilirating atmosphere, and the number and variety of its medicinal springs, is believed to be unexcelled by any portion of the globe. If this anticipation proves true there will be reason to be gratified at the wise forethought which has secured forever as a national park this magnificent domain, extending nearly sixty-five miles from north to south and fifty-five miles from east to west. It is under the exclusive control of the Secretary of the Interior, who may at his discretion grant leases, for terms not exceeding ten years, of small parcels of ground at such places as shall require the erection of buildings for the accommodation of visitors, all the proceeds of said leases to be expended under his direction in the construction of roads, etc., etc. The altitude of this reservation is over 6,000 feet, and the mountains that hem the valleys in on every side rise to the height of 10,000 to 12,000 feet, and are covered with snow all the year. These mountains are all of volcanic origin, and probably contain no valuable minerals. During the months of June, July and August the climate is pure and invigorating, with scarcely any rain or storms of any kind; but the thermometer frequently sinks as low as 26°. There is frost every month of the year.

The Yellowstone lake occupies an area of fifteen by twenty-two miles, or 330 square miles, and its altitude is 7,427 feet. It abounds in salmon trout, and is visited by great numbers of wild fowl.

The Yellowstone River flows from Yellowstone Lake almost due north. It is a tributary of the Missouri. Its length is 1,300 miles. The upper portion of the river flows through immense cañons and gorges, with waterfalls and rapids. The entire region abounds in boiling springs, mud volcanos, soda springs, sulphur mountains, and geysers more wonderful than those of Iceland. It is claimed that in no other portion of the globe are there united so many surprising features.

### THE HOT SPRINGS.

Upon the west side of Gardiner's river, upon the slope of the mountain, is one of the most remarkable groups of hot springs in the world. The springs in action at the present time are not very numerous, or even so wonderful as some of those higher up the Yellowstone Valley or in the Fire Hole Basin. The calcareous deposits from these springs cover an area of about two square miles. The active springs extend from the margin of the river 5,545 feet to an elevation of 6,522 feet above the sea.

### WHITE MOUNTAIN HOT SPRING

is located on the side of the mountain. The snowy whiteness of the deposit gives it the appearance of a frozen cascade. If a group of springs near the summit of a mountain were to distribute their waters down the irregular declivities, and they were slowly congealed, the picture would bear some resemblance in form. Here is a hill, 200 feet high, composed of the calcareous deposit of the hot springs, with a system of step-like terraces which defy description. The steep sides of the hill are ornamented with a series of semicircular basins, with margins varying in height from a few inches to six or eight feet, and so beautifully scalloped and adorned with a kind of bead work, that the beholder stands amazed at this marvel of Nature's handiwork. Add to this a snow white ground, with every variety of shade of scarlet, green and yellow, as brilliant as the brightest of our aniline dyes. As the water flows from the spring over the mountain side, from one basin to another, it loses continually a portion of its heat, and the bather can find any desirable temperature. On the top of the hill there is a broad, flat terrace, covered more or less with these basins, 150 to 200 yards in diameter. We here find the largest and most active spring at the present time. It is very near the outer margin of the terrace, and is 25 by 40 feet in diameter, the water so transparent that one can look down to the bottom of the basin. The sides of the basin are ornamented with coral like forms with a great variety of shades, from pure white to bright cream yellow, and the blue sky reflected in the waters gives an azure tint to the whole which surpasses all art. Underneath the sides of many of these pools are rows of stalactites of all sizes, many of them exquisitely ornamented, formed by the dripping of the water over the margins of the basins. On the west side of this deposit old chimneys or craters are scattered thickly over the surface, and there are several large holes and fissures leading to vast caverns beneath the crust. The entire Yellowstone basin is covered more or less with dead and dying springs, but there are centres or groups where the activity is greatest at the present time. Below the falls there is an area of ten or fifteen square miles covered with deposits. On the south side of Mount Washburn there is a group of active springs; sulphur, copper, alum and soda cover the surface. These springs are located on the side of the mountain, nearly 1,000 feet above the margin of the cañon, but extend along into the level portion below. Extending across the cañon, on the opposite side of the Yellowstone, this group of springs extends for several miles. Many of the dead springs are mere basins, with a thick deposit of iron on the sides, lining the channel of the water that flows from them. These vary in temperature from 98° to 120°. The highest temperature is 192°. The steam vents are numerous, and the chimneys are lined with sulphur.

### MUD SPRINGS.

These springs are scattered along on both sides of the river, sometimes extending upon the hillsides 50 to 200 feet above the river. The first one is a remarkable mud spring, with a well defined circular rim composed of fine clay, and raised about four feet above the surface around, and about six feet above the mud in the basin. The diameter of the basin is about eight feet. The mud is so fine as to be impalpable, and the whole may be most aptly compared to a caldron of boiling mush. The gas is constantly escaping, throwing up the mud from a few inches to six feet in height. About twenty yards distant from the mud spring just described is a second one, with a basin nearly circular forty feet in diameter, the water six or eight feet below the margin of the rim. The water is quite turbid, and is boiling moderately. Small springs are flowing into it from the south side, so that the basin forms a sort of reservoir. The temperature in some portions of the basin is thus lowered to 98°. Several small hot springs pour their surplus water into it, the temperature of which are 180°, and 170°, and 184°, and 155°. In the reservoir, where the water boils up with considerable force, the temperature is only 96°, showing that the bubbling was due to the escape of gas.

About twenty feet from the last is a small mud spring with an orifice ten inches in diameter, with whitish brown mud, 182°. Another basin, near the last, has two orifices, the one throwing out mud with a dull thud about once in three seconds, spurting the mud three or four feet; the other is content to boil up quite violently, occasionally throwing the mud ten or twelve inches. This mud, which has been wrought in these caldrons for perhaps hundreds of years, is very fine and pure. The contents of many of the springs are of such snowy whiteness that, when dried in cakes in the sun or by a fire, they resemble the finest meerschaum. The color of the mud depends upon the superficial deposits which cover the ground through which the waters of the spring reach the surface. They were all clear hot springs originally—perhaps geysers even; but the continual caving in of the sides has produced a sort of mud pot, exactly the same as the process of preparing a kettle of mush. The water is at first clear and hot; then it becomes turbid from the mingling of the loose earth around the sides of the orifice until, by continued accessions of earth, the contents of the basin becomes of the consistency of thick mush. Every possible variation of condition of the contents is found, from simple milky turbiness to a stiff mortar. On the east side of the Yellowstone, close to the margin of the river, are a few turbid and mud springs strongly impregnated with alum. The mud is quite yellow, and contains much sulphur. The basin is fifteen by thirty feet, and has three centres of ebullition, showing that, deep down underneath the superficial earth, there are three separate orifices, not connected with each other, for the emission of heated waters.

### SULPHUR MOUNTAIN AND MUD VOLCANO.

Toward the western verge of a prairie of several miles in extent, above the Yellowstone Falls, a hill of white rock was discovered, which, upon investigation, proved to be another of the soda mountains, as they are called by the hunters. Jets of smoke and steam issue from the face of the hill, while its other side is hollowed out into a sort of amphitheatre, whose sides steam with sulphur fumes, the ground hot and parched with internal fires. There are acres of this hot volcanic surface, having numerous cracks and small apertures at intervals of a few feet, whence are expelled, sometimes in steady continuous streams sometimes in puffs like those of an engine, jets of vapor more or less impregnated with mineral substances. The thin rock crust, which in many places gives way beneath the tread, reveals caverns of pure crystallized sulphur from which hot fumes issue. A large spring, emitting strong fumes of sulphur and sulphuretted hydrogen not at all agreeable, was also found. Upon the opposite side a number of large springs has been found—one, from its size and its power in throwing water to the height of several feet above the surface, is worthy of notice. Near this is a spring having regular pulsations, like a steam engine, giving off large quantities of steam which issues forth with the roar of a hurricane. This is, in reality, a steam volcano ; deep vibrations in the subterraneous caverns can be distinctly heard. The country from this point to the mud volcano, a few miles above, is mostly rolling prairie, intersected with several streams flowing into the river, some of them having wide estuaries and adjacent swampy flats, covered with thick marsh grass. Ducks are usually found in these sluggish streams, as well as in the little lakes so numerous throughout this region.

THE MUD GEYSER

is near the bank of the river. It has broken out from the side of a well timbered hill. The crater is twenty five feet across at top, gradually sloping inward to the bottom where it becomes about half this diameter. Its depth is about thirty feet. The deposit is gray mud, nearly pure alumina, and has been thrown up by the action of the volcano at no very distant period. The rim of the crater, on the down hill side, is some ten feet in length, and trees fifty feet high and a hundred feet distant are loaded with mud thrown from this volcano. The surface of the bottom is in a constant state of ebullition, puffing and throwing up masses of boiling mud, and sending forth dense columns of steam several hundred feet above the surrounding forests. This vapor can be seen for many miles in all directions. Some four hundred yards from this crater are three large hot springs of muddy water, one of which proved to be a geyser, having periods of active eruption about every six hours. The phenomina attending these eruptions are as follows: Soon after the violent period passes, the water in the pool gradually subsides through the orifice in the centre, the surface falling several feet, the water almost entirely disappearing from sight. It then gradually rises again until the former level is reached, during which occasional ebullitions of greater or lesser magnitude occur. Great agitation then ensues; pulsations, at regular intervals of a few seconds take place, at each of which the water in the crater is elevated higher and higher until, finally, after ten minutes, a column is forced up to the height of thirty or forty feet. During this period waves dash against the sides of the basin, vast clouds of steam escape, and a noise like the rumbling of an earthquake takes place. Suddenly, after about fifteen minutes of this commotion, the waves recede, quiet is restored, the waters sink gradually to their lowest limit, from which they soon rise again and repeat the same operation.

THE GREAT GEYSER BASIN.

Entering the basin from the north, and following the banks of the Fire Hole River, whose direction there is about northeast, a series of rapids, quite near together, is encountered, when the river makes a sharp bend to the southwest, at which point is found a small steam jet upon the right. A warm stream comes in from the left, falling over a bank ten feet in height. A short distance beyond a second rapid is found, and then another about 100 yards further on, where the gate of the GEYSER BASIN is entered. Here, on either side of the river, are two lively geysers called the SENTINELS. The one on the left is in constant agitation, its waters revolving horizontally with great violence, and occasionally spouting upward to the height of twenty feet, the lateral direction being fifty feet. Enormous masses of steam are ejected. The crater of this geyser is three feet by ten. The opposite SENTINEL is not so active, and is smaller. The rapids here are 200 yards in length, with a fall of 30 feet. Following the bank of the river, whose general course is from the southeast, though with many windings, 250 yards from the gate are three geysers acting in concert. When in full action the display from these is very fine. The waters spread out in the shape of a fan, in consequence of which they have been named the FAN GEYSERS. A plateau, opposite the latter, contains fifteen hot springs, of various characteristics. Some are of a deep blue color, from sulphate of copper held in solution, and having fanciful caverns distinctly visible below the surface of the water. The openings at the surface are often beautifully edged with delicately wrought fringes of scalloped rock. One variety deposits a red or brown leathery substance, partially adhering to the sides and bottom of the cavern, and waving to and fro in the water like plants. The size of these springs varies from five to forty feet in diameter. One hundred yards farther up the east side of the stream is found a double geyser, a stream from one of its orifices playing to the height of eighty or ninety feet, emitting large volumes of steam. From the formation of its crater it was named the WELL GEYSER. Above is a pine swamp of cold water, opposite which, and just above the plateau previously mentioned, are found some of the most interesting and beautiful geysers of the whole basin. First we come upon two smaller geysers near a large spring of blue water, while a few yards beyond are seen the walls and arches of the GROTTO. This is an intricate formation eight feet high and ninety in circumference. It is hollowed into fantastic arches, with pillars and walls of almost indescribable variety. This geyser plays to the height of sixty feet several times during twenty-four hours. The water, as it issues from its numerous apertures, has a very striking and picturesque effect. Near the GROTTO is a large crater, elevated four feet above the surface of the hill, having a rough shaped opening measuring two by two and a half feet. Two hundred yards farther up are two very fine large geysers, between which and the GROTTO are two boiling springs. Proceeding 150 yards farther, and passing two hot springs, a remarkable group of geysers is discovered. One of these has a huge crater five feet in diameter, shaped something like the base of a horn—one side broken down—the highest point being fifteen feet above the mound on which it stands. This is a tremendous geyser, called the GIANT. It throws a column of water the size of the opening to the height of 130 feet, and continues the display for an hour and a half. The amount of water discharged is immense—about equal in quantity to that in the river, the volume of which during the eruption is doubled.

We have given a description of all the most prominent features of this wonderful region, which we trust will be read with interest, not by invalids alone, but by all admirers of the beautiful, the grand and the marvellous in nature. Among the hundreds of springs in the YELLOWSTONE REGION is found every variety of temperature and of chemical analysis. It is probable that every celebrated spring in the world may have a duplicate here. Unfortunately, this region is not at present easily accessible, and we are not aware of the existence of hotels or other accommodations for visitors; but the time is probably not distant when these requisites will be provided, and we predict that it will then become the most important and the most popular HEALTH RESORT on this continent.

